# 1,25-Dihydroxyvitamin D_3_ Suppresses UV-Induced Poly(ADP-Ribose) Levels in Primary Human Keratinocytes, as Detected by a Novel Whole-Cell ELISA

**DOI:** 10.3390/ijms25115583

**Published:** 2024-05-21

**Authors:** Warusavithana Gunawardena Manori De Silva, Vanessa Bernadette Sequeira, Chen Yang, Katie Marie Dixon, Andrew J. A. Holland, Rebecca Sara Mason, Mark Stephen Rybchyn

**Affiliations:** 1 Department of Physiology, School of Medical Sciences and Bosch Institute, The University of Sydney, Sydney, NSW 2006, Australia; 2 Department of Anatomy and Histology and Bosch Institute, The University of Sydney, Sydney, NSW 2006, Australia; katie.dixon@sydney.edu.au; 3 Douglas Cohen Department of Paediatric Surgery, The Children’s Hospital at Westmead Clinical School, The Faculty of Medicine and Health, The University of Sydney, Sydney, NSW 2006, Australia; 4 School of Life and Environmental Sciences and Charles Perkins Centre, The University of Sydney, Sydney, NSW 2006, Australia

**Keywords:** 1,25-dihydroxyvitamin D_3_ (1,25(OH)_2_D_3_), 3-aminobenzamide (3AB), 8-hydroxy-2′-deoxyguanosine (8-OHdG), cyclobutane pyrimidine dimer (CPD), enzyme-linked immunosorbent assay (ELISA), extra-cellular regulated kinase (ERK), poly(ADP-ribose) (pADPr), poly(ADP-ribose) polymerase-1 (PARP-1), nicotinamide adenine dinucleotide (NAD+), ultraviolet irradiation (UVR)

## Abstract

Photoprotective properties of 1,25-dihydroxyvitamin D_3_ (1,25(OH)_2_D_3_) to reduce UV-induced DNA damage have been established in several studies. UV-induced DNA damage in skin such as single or double strand breaks is known to initiate several cellular mechanisms including activation of poly(ADP-ribose) (pADPr) polymerase-1 (PARP-1). DNA damage from UV also increases extracellular signal-related kinase (ERK) phosphorylation, which further increases PARP activity. PARP-1 functions by using cellular nicotinamide adenine dinucleotide (NAD+) to synthesise pADPr moieties and attach these to target proteins involved in DNA repair. Excessive PARP-1 activation following cellular stress such as UV irradiation may result in excessive levels of cellular pADPr. This can also have deleterious effects on cellular energy levels due to depletion of NAD+ to suboptimal levels. Since our previous work indicated that 1,25(OH)_2_D_3_ reduced UV-induced DNA damage in part through increased repair via increased energy availability, the current study investigated the effect of 1,25(OH)_2_D_3_ on UV-induced PARP-1 activity using a novel whole-cell enzyme- linked immunosorbent assay (ELISA) which quantified levels of the enzymatic product of PARP-1, pADPr. This whole cell assay used around 5000 cells per replicate measurement, which represents a 200–400-fold decrease in cell requirement compared to current commercial assays that measure in vitro pADPr levels. Using our assay, we observed that UV exposure significantly increased pADPr levels in human keratinocytes, while 1,25(OH)_2_D_3_ significantly reduced levels of UV-induced pADPr in primary human keratinocytes to a similar extent as a known PARP-1 inhibitor, 3-aminobenzamide (3AB). Further, both 1,25(OH)_2_D_3_ and 3AB as well as a peptide inhibitor of ERK-phosphorylation significantly reduced DNA damage in UV-exposed keratinocytes. The current findings support the proposal that reduction in pADPr levels may be critical for the function of 1,25(OH)_2_D_3_ in skin to reduce UV-induced DNA damage.

## 1. Introduction

Poly(ADP-ribose) polymerase (PARP) is a ubiquitous nuclear enzyme that was reported as early as 1963 [1]. It is found in mammalian and some eukaryotic cells [2,3,4]. Eighteen different protein isoforms have been identified [5]. PARP-1 is the most prominent member of the PARP family, a 116 kDa zinc-finger nuclear protein that is involved in DNA repair due to environmental stress. PARP-1 binds to DNA single- or double- stranded breaks and then PARylates acceptor proteins (including itself) [6,7]. While DNA damage directly increases PARP-1 activity, there is evidence for further increased PARP activation after UV as a result of increased extracellular signal-regulated kinase (ERK) phosphorylation [8,9,10,11]. PARP has three highly conserved domains: an amino-terminal, DNA-binding domain (binds to the damaged DNA and causes conformational shift) [12,13,14,15]; a central auto-modification domain (releases proteins from DNA) [15,16,17]; and a carboxyl-terminal, catalytic domain [15,18,19]. PARP-1 is a substrate for cellular caspases [20], which facilitate apoptosis. PARP-1 is proteolysed by caspase-3 and -7 into an 89 kDa catalytic domain and a 24 kDa DNA-binding domain [12,21,22,23,24]. This cleavage suppresses PARP-1 activity [21,25] as binding of the 24 kDa fragment alone to the damaged DNA site inhibits repair due to the lack of the catalytic fragment [26].

Mild DNA damage initiates DNA repair pathways, but if the damage is severe, the cell will undergo apoptosis or necrosis. PARP-1 is activated upon DNA damage recognition, and activated PARP utilizes cellular nicotinamide adenine dinucleotide (NAD+) to form nicotinamide and ADP-ribosyl moieties (PARs) into protein acceptor molecules (histones, transcription factors) to form long-chain poly(ADP-ribose) (pADPr), a process known as PARylation. The removal of pADPr is mainly carried out by poly(ADP-ribose) glycohydrolase (PARG), without which there is uncontrolled pADPr accumulation and cytotoxicity [27]. Excessive PARylation depletes cellular NAD+ and energy levels [28,29,30,31,32,33] ultimately, if not controlled, leading to “parthanatos”, a distinct type of cellular death that occurs due to excessive PARP-1 activation [24]. It has also been reported that energy depletion by activated PARP-1 is due to inhibition of the rate-limiting enzyme, hexokinase, in glycolysis [34,35]. A combination of these factors and mitochondrial damage by UV, which increases oxidative stress, leads to depletion of cellular NAD+ and adenosine triphosphate (ATP) levels that may contribute to reduced energy levels which inhibit energy-demanding cellular repair processes and thereby may progress the cell to parthanatos, apoptosis, or necrosis. Although not the focus of this study, it should be noted that PARP-1 function is closely linked with poly(ADP-ribose) glycohydrolase (PARG) activity, an enzyme which catalyses the breakdown of PARs formed by PARP proteins. This process ultimately recycles energy back into the cellular system [36], and several lines of evidence suggest that PAR removal is required for efficient DNA repair [36,37,38].

The active vitamin D hormone, 1,25-dihydroxyvitamin D_3_ (1,25(OH)_2_D_3_), which is made in skin as a result of UVB exposure [39,40,41,42], has been shown to reduce UV-induced DNA damage, both cyclobutane pyrimidine dimers (CPD) [43] and oxidative DNA damage, measured as 8-hydroxy-2′-deoxyguanosine (8-OHdG) [44,45,46,47] in human keratinocytes [48,49,50], ex vivo in human skin explants [46] and in vivo in Skh:hr1 mice [43,48,49,50,51,52,53] and in human volunteers [54]. At least part of its mechanism of action is to increase DNA repair through an increase in glycolysis with concurrent suppression of energy consuming anabolic cellular signalling pathways ultimately leading to increased cellular ATP levels [45].

Given that UV exposure greatly increases PARP-1, which in turn inhibits glycolysis [34,35], we previously speculated that reduced accumulation of pADPr moieties was important in the mechanism of action of 1,25(OH)_2_D_3_, but had no way to test this given the large cell numbers required for commercial PARP assays available at the time. The current study investigated the effect of 1,25(OH)_2_D_3_ on UV-induced PARP in primary human keratinocytes using a novel whole-cell ELISA to quantify the enzymatic product, pADPr. Because UV also increases phosphorylation of ERK (ERK-p) [55], which increases PARP-1 activity [8,9,11], and we have reported that 1,25(OH)_2_D_3_ reduced UV-induced ERK-p [45], we examined whether the known PARP inhibitor, 3AB, as well as a peptide inhibitor of ERK-p would mimic the effects of 1,25(OH)_2_D_3_ on UV-induced DNA damage.

## 2. Results

### 2.1. UV Dose–Response Study to Detect Phosphorylated PARP Product, pADPr with a Novel Whole-Cell ELISA

Whole-cell enzyme linked immunosorbent assay (ELISA) or cell-based ELISA is an immunocytometric technique used to target and identify cellular proteins or post-translational modifications of target protein in cultured cells. It is a sensitive and a versatile technique [56]. For the novel assay, described in more detail in Methods, keratinocytes were fixed with formaldehyde 5 or 15 min after irradiation and then permeabilised. They were incubated with an antibody to pADPr or an isotype control. After overnight incubation at 4 °C, the cells were incubated with anti-mouse IgG horse radish peroxidase (HRP)-linked antibody, followed by the addition of HRP substrate. Absorbance was measured at 450 nm.

**Figure 1:**
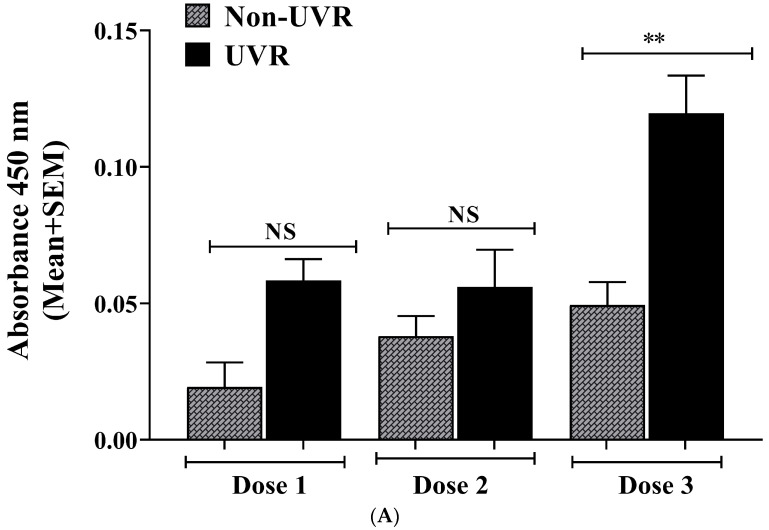

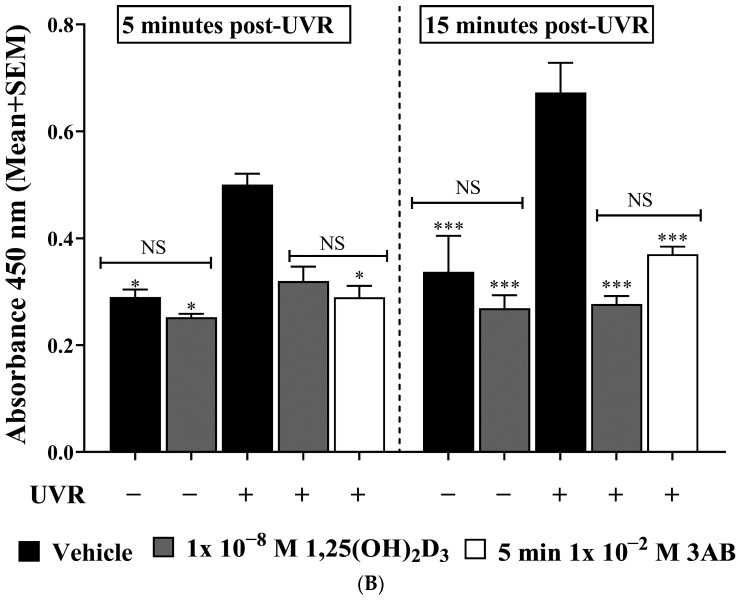
(**A**) UV dose response for ssUV-induced pADPr in human primary keratinocytes. Human primary keratinocytes were exposed to solar-simulated UV as described in Methods. Dose 1 = 400 mJ/cm^2^ UVB and 3600 mJ/cm^2^ UVA, equivalent to 4 min of noon-day sun in Sydney, Australia in October (Doses 2 and 3 were 2-fold and 3-fold dose 1 respectively). pADPr were measured by a whole-cell ELISA by absorbance at 450 nm. Significant increases in UV-induced pADPr compared to non-UV were not detected at UV doses 1 and 2, but at dose 3 (** *p* < 0.01), 15 min post-UV. Results from a single experiment, performed in triplicate, representative of 4 independent experiments at the highest UV dose, each with similar results. Means + SEM; ** *p* < 0.01 when compared to UV vehicle; NS—not significant between data sets. (**B**) ssUV-induced pADPr levels after 5 or 15 min and reduction in pADPr activity by treatment with 1,25(OH)_2_D_3_ or PARP inhibitor 3-aminobenzidine (3AB). UV-exposed keratinocytes treated with vehicle [0.1% (*v*/*v*) Etoh], 1 × 10^−8^ M 1,25(OH)_2_D_3_ or 1 × 10^−3^ M 3AB concentrations, fixed 5 or 15 min after UV. Results are from a single experiment performed in triplicate, representative of 4 separate experiments with similar results. Means + SEM; * *p* < 0.05, when compared to 5 min UV vehicle; *** *p* < 0.001 when compared to 15 min UV vehicle; NS—not significant between datasets.

Whereas a solar-simulated UV dose equivalent to 4 min exposure to noon-day sun in October in Sydney, Australia (Dose 1) has generally been used on human keratinocytes for DNA damage studies by our group [45], this dose was insufficient to cause measurable UV-induced increases to the levels of pADPr compared to non-irradiated cells (Figure 1A). Increasing the UV irradiation dose three-fold (Dose 3) resulted in significant increases to UV-induced pADPr compared to non-irradiated cells from 0.05 ± 0.01 absorbance units (AU) to 0.12 ± 0.01 (AU) (Figure 1A, *p* < 0.01). Thus, Dose 3 of UV (equivalent to 12 min of noon-day sun in October in Sydney, Australia) was used for the ELISA experiments.

### 2.2. UV-Induced pADPr Levels Were Significantly Reduced by Treatment with 1,25(OH)_2_D_3_ or 3AB in Human Primary Keratinocytes

Significant increases in UV-induced pADPr were detected at 5 min from 0.3 ± 0.01 AU to 0.5 ± 0.02 AU (Figure 1B, *p* < 0.05) and 15 min post-UV from 0.3 ± 0.07 AU to 0.7 ± 0.06 AU (Figure 1B, *p* < 0.001) compared to non-irradiated cells. Treatment with 1,25(OH)_2_D_3_ resulted in significant suppression of pADPr levels 15 min post-irradiation from 0.7 ± 0.06 AU to 0.3 ± 0.01 AU (Figure 1B, *p* < 0.001) with a non-significant decrease at 5 min post-irradiation (Figure 1B, NS). The addition of the known PARP-1 inhibitor 3-aminobenzimide (3AB) [57] resulted in decreased pADPr levels after UV from 0.5 ± 0.02 AU to 0.3 ± 0.02 AU at 5 min (Figure 1B, *p* < 0.05) and from 0.7 ± 0.06 AU to 0.4 ± 0.01 AU 15 min post-UV (Figure 1B, *p* < 0.001), not significantly different from those in cells treated with 1,25(OH)_2_D_3_ (Figure 1B).

### 2.3. Treatment of UV-Irradiated Keratinocytes with 1,25(OH)_2_D_3_ or 3AB Resulted in a Significant Reduction in CPD Levels

**Figure 2:**
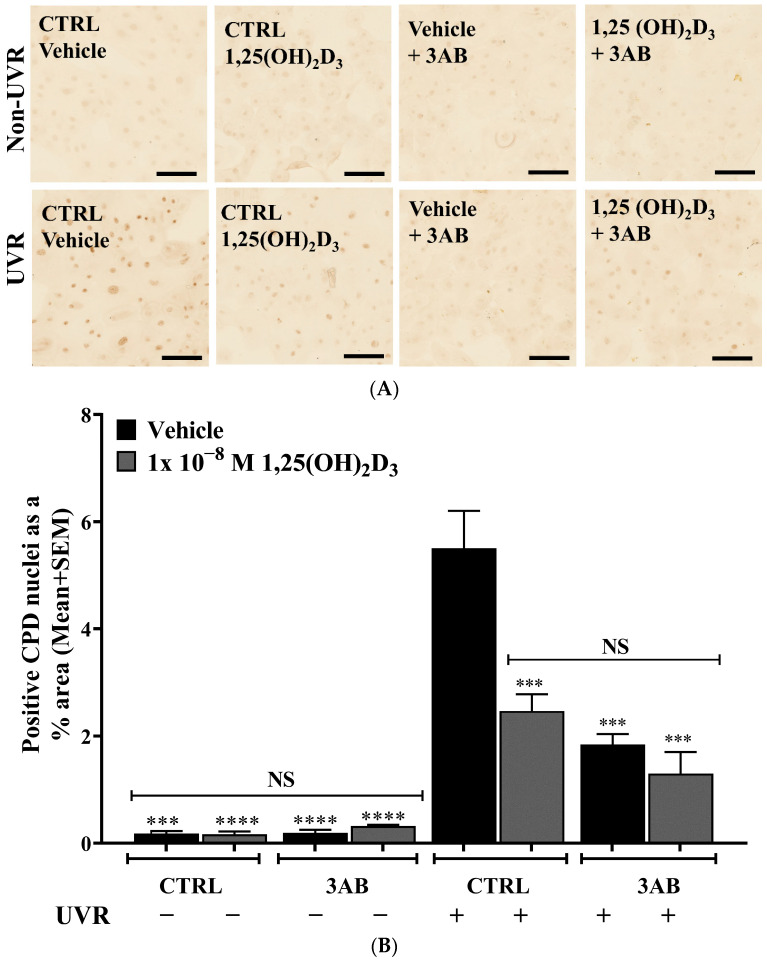

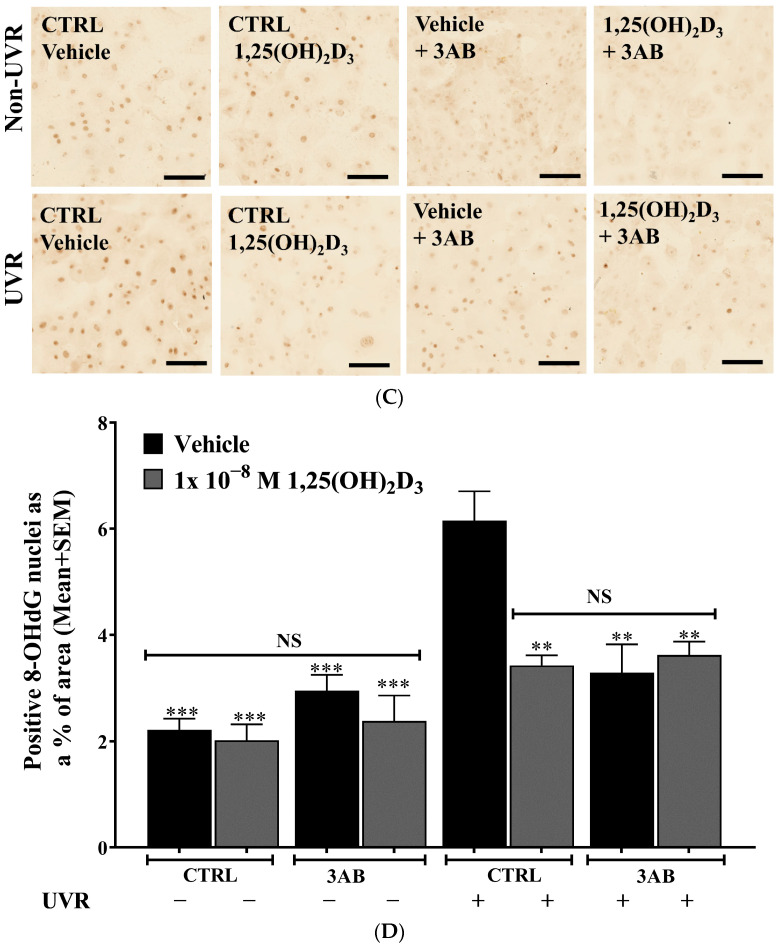
Both 1,25(OH)_2_D_3_ and 3AB reduced UV-induced DNA damage. Example bright field confocal IHC photomicrographs of (**A**) UV-induced CPD or (**C**) UV-induced 8-OHdG in keratinocytes treated with vehicle [0.1% (*v*/*v*) Etoh], 1 × 10^−8^ M 1,25(OH)_2_D_3_ or 1 × 10^−3^ M 3AB or a combination of these at the aforementioned concentrations, fixed 1.5 h after UV for immunohistochemical staining (scale bar = 100 µm). (**B**) Densitometry presented as CPD-positive nuclei as a % area. (**D**) 8-OHdG positive nuclei as a % area. Results are from a single experiment performed in triplicate, representative of 3 separate experiments with similar results. Means + SEM; ** *p* < 0.01; *** *p* < 0.001; **** *p* < 0.0001, when compared to UV vehicle; NS—not significant between data sets.

UV irradiation of primary human keratinocytes resulted in a significant ~31-fold increase in detected CPD levels from 0.2 ± 0.05% area to 5.5 ± 0.7% area (Figure 2A,B, *p* < 0.0001), which is generally considered a marker of direct DNA damage by UV [58]. Treatment of keratinocytes with 1,25(OH)_2_D_3_ resulted in a significant ~50% reduction in detected CPD levels compared to vehicle-treated cells (Figure 2B, *p* < 0.001). Treatment of keratinocytes with 3AB also resulted in a similar significant ~60% reduction in detected CPD levels compared to vehicle-treated cells (Figure 2B, *p* < 0.001). The combination of 1,25(OH)_2_D_3_ and 3AB produced no further decrease in CPD.

### 2.4. Treatment of UV-Irradiated Keratinocytes with 1,25(OH)_2_D_3_ or 3AB Resulted in a Significant Reduction in 8-OHdG Levels

UV irradiation of primary human keratinocytes resulted in a significant ~3-fold increase in detected 8-OHdG levels from 2.2 ± 0.2% area to 6.2 ± 0.5% area (Figure 2C,D, *p* < 0.001), a marker of oxidative DNA damage [59,60,61,62,63]. Treatment of keratinocytes with 1,25(OH)_2_D_3_ resulted in a significant ~50% reduction in detected 8-OHdG levels compared to vehicle-treated cells (Figure 2D, *p* < 0.01). Treatment of keratinocytes with 3AB also resulted in a similar significant ~50% reduction in detected 8-OHdG levels compared to vehicle-treated cells (Figure 2D, *p* < 0.01). Again, the combination of 1,25(OH)_2_D_3_ and 3AB produced no further decrease in 8-OHdG.

### 2.5. A Peptide ERK Inhibitor Reduced UV-Induced CPD Levels to a Similar Extent as 1,25(OH)_2_D_3_

**Figure 3:**
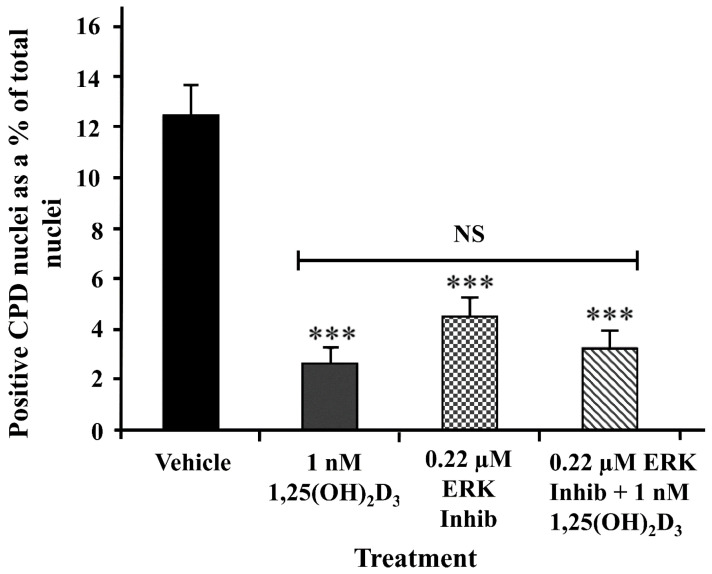
UV-induced DNA damage was reduced by both 1,25(OH)_2_D_3_ and an ERK peptide inhibitor in human keratinocytes. UV-induced CPD detected by immunohistochemistry 3 h after UV was quantified by image analysis and expressed as positively stained nuclei as a proportion of total nuclei. Keratinocytes were treated with vehicle [0.1% (*v*/*v*) Etoh], 0.22 µM ERK peptide inhibitor II or 1 nM 1,25(OH)_2_D or a combination of 1,25(OH)_2_D and the ERK peptide inhibitor at the aforementioned concentrations. Pooled data from a minimum of three independent experiments; means + SEM. Significantly different from vehicle, *** p < 0.001. NS—not significant between data sets.

PARP activity has previously been shown to be increased via direct interaction with phosphorylated ERK_1/2_ [10] as well as by direct DNA damage [6,7]. We previously reported that 1,25(OH)_2_D_3_ treatment of primary human keratinocytes reduced phosphorylation of ERK [45]. To provide a possible mechanistic explanation as to how 1,25(OH)_2_D_3_ suppresses PARP-1 activity in keratinocytes, we examined the effect of treatment of keratinocytes with ERK activation inhibitor peptide II (IC_50_ = 0.21 µM). This ERK inhibitor reduced UV-induced CPD from 12.5 ± 0.5% area to 4.7 ± 0.3% area, similar to the effect of 1,25(OH)_2_D_3_ to 2.7 ± 0.2% area (Figure 3), and there was no additional effect when the two agents were used together.

## 3. Discussion

Using a novel whole-cell ELISA, which required only ~5000 cells per replicate measurement, we were able to reliably quantify PARP activity as defined by the presence of the enzymatic product pADPr in the cells [64]. Commercially available PARP activity assays require 10^6^–10^7^ cells per treatment [65]. Due to the limited number of primary keratinocytes from a single donor and the limit of cell division cycles by keratinocytes in vitro [66] generating such large numbers of cells is not technically feasible. Furthermore, the formalin-based fixed cell assay used in this study did not require a lysis step, which may possibly allow specific (i.e., PARG) or non-specific breakdown of pADPr moieties prior to analysis. Our research group has previously used a similar type of assay to successfully measure UV-induced 3-nitrotyrosine levels in this cell phenotype [48].

Excessive activation of PARP-1 has been shown to result in depletion of cellular energy levels via NAD+ consumption [28,29,32], and due to inhibition of the rate-limiting enzyme, hexokinase, in glycolysis [34,35]. Suppressed PARP-1 activity by 1,25(OH)_2_D_3_ would in turn result in less UV-induced suppression of hexokinase activity, which would reduce UV-induced glycolytic blockade in the irradiated cell [34]. Reducing the depletion of NAD+ and conserving cellular energy pools [28,31,32] would contribute to increased repair of DNA damage. These results extend and complement earlier studies by our group showing that control of pathways and processes that regulate cellular energy is critical for repair of UV-induced DNA damage in skin cells [45]. UV-induced PARP-1 activity was shown to be reduced by treatment with 1,25(OH)_2_D_3_ in the immortalised keratinocyte HaCaT cell line [67,68]. Our group has previously shown that increased glycolysis and suppression of cellular growth pathways (e.g., mTOR) are observed following treatment with 1,25(OH)_2_D_3_ in primary human keratinocytes, ultimately leading to increased cellular ATP levels [45]. Such an energetically favorable outcome would be expected to be observed if UV-induced glycolytic blockade was alleviated in response to 1,25(OH)_2_D_3_ treatment. Therefore, the data presented here further reveal the role that 1,25(OH)_2_D_3_ plays in post-irradiation control of cell energy levels, via suppression of excessive accumulation of pADPr [69]. In vivo, it is likely that 1,25(OH)_2_D_3,_ which is produced and accumulates in the skin after UV exposure [39,40], may in part contribute to increases in cellular energy levels and thus act as a defense mechanism against subsequent rounds of UV exposure by permitting the cell to more effectively repair UV-induced damage.

Actions of 1,25(OH)_2_D_3_ to reduce keratinocyte losses after UV and to inhibit oxidative stress, either measured as oxidative UV damage or other oxidative or nitrosative stress markers, have been reported in human keratinocytes and in mouse skin [45,51,70]. Indeed, patients with non-melanoma skin cancers and mostly low vitamin D status had higher markers of oxidative stress in their blood than non-skin cancer controls [71]. The combination of increased DNA repair, together with reduced oxidative stress in the presence of 1,25(OH)_2_D_3_, may explain the higher burden of non-melanoma skin cancers observed in mice with knockout of the vitamin D receptor after chemical or chronic UV treatments [72,73] and the reduced skin cancers reported in mice exposed to long-term UV but treated topically with 1,25(OH)_2_D_3_ [51]. It thus seems likely that 1,25(OH)_2_D_3_ and possibly other related compounds made in skin as a result of UV exposure [39,40,41,42] result in less skin damage than would otherwise be the case, although this is difficult to test in human populations.

Previously published studies have shown that PARP activity can be controlled by phosphorylated ERK [8,9,64,74,75,76]. As previously reported, 1,25(OH)_2_D_3_ reduced UV-induced phosphorylated ERK [45], thus providing a possible molecular mechanism for how 1,25(OH)_2_D_3_ may suppress PARP activity, since this inhibition of ERK phosphorylation would then result in decreased accumulation of pADPr moieties, as observed. In support of the proposal that increased PARP-1 activity leads to reduced energy for DNA repair, we observed that a peptide inhibitor of ERK phosphorylation and a direct inhibitor of PARP-1 [77], 3AB, both inhibited UV-induced DNA damage to a similar extent as 1,25(OH)_2_D_3_ and that there was no additive effect of 1,25(OH)_2_D_3_ with either agent.

In skin cells, 1,25(OH)_2_D_3_ is generally thought to function via two separate but probably complementary pathways: (1) the classical steroid receptor/genomic pathway, which involves VDR/RXR-dependent (vitamin D receptor-retinoid X receptors) gene transcription following translocation of the receptor complex from the nuclear membrane and (2) the rapid response “non–genomic” pathway, which is also thought to act via the VDR that is non-nuclear-associated [70]. The rapid suppression of ERK-p by 1,25(OH)_2_D_3_ might support a non-genomic mechanism. Other evidence also points to 1,25(OH)_2_D_3_-dependent suppression of PARP-1 activity in a variety of model systems via both the genomic and non-genomic pathways. In a diabetic rat model, increased *parp-1* gene expression was significantly reduced in animals that were on a vitamin D_3_ diet [78]. In a separate diabetic rat model, direct oral administration of 1,25(OH)_2_D_3_ resulted in decreased PARP-1 quantified at the protein level [79]. In a study of Parkinson’s disease (PD) pathophysiology, 1,25(OH)_2_D_3_ relieved parthanatos in a neuroblastoma cellular model [80]. This study also showed a direct interaction between PARP-1 and the VDR, and overexpression of VDR in this cellular model reduced PARP-1 protein levels [80]. Another mechanistic possibility is that, given pADPr moieties need to be removed by PARG prior to DNA repair [36,69], 1,25(OH)_2_D_3_ may facilitate quicker removal of pADPr groups, thereby allowing the DNA repair machinery faster and better access to the DNA lesion for repair [27,81]. Our assay, which measures these pADPr moieties, does not distinguish between inhibited PARP activity or increased removal of pADPr. Other limitations of the study are that we needed three times the dose of solar-simulated UV to show a significant increase in pADPr compared to that used for demonstration of DNA damage. Although this is hardly excessive, being the equivalent of 12 min of noon day sun in Sydney, Australia in October, it suggests that the assay is not as sensitive as other immunohistochemical methods. Also limiting is that the studies, for practical reasons, were carried out in cell culture and not in whole skin.

Since PARP is known to contribute to the DNA repair process [11,27], why PARP inhibition would reduce UV-induced DNA lesions following a non-lethal dose of UV radiation is unclear. It is becoming increasingly accepted, however, that the balance between pADPr synthesis and removal is key to optimal repair [69]. It is also plausible that in the absence of PARP activation the cells may shift to other repair mechanisms, or energy-consuming functions, that would otherwise be suppressed. There is a wide range of examples in the literature where PARP inhibitors exert beneficial effects [82,83,84], but several other studies provided contradictory findings [85,86,87]. In UV-irradiated normal human epidermal keratinocytes treated with 3AB, reduced necrosis, apoptosis, and ROS production were reported [83]. *PARP*^−/−^ mice were reported to be viable and fertile but with increased susceptibility to skin disease in older mice [88]. Further, the cells from *PARP*^−/−^ mice maintained efficient repair of damaged DNA [88,89,90,91,92]. It is also possible that excessive PARP-1 activation may result in excessive accumulation of pADPr moieties which inhibit repair. Topical application of a PARP inhibitor, BPG-15M [*O*-(3-pyperidino-2-hydroxy-1-propyl) pyridine-3-carboxylic acid amidoxime monohydrochloride], to hairless mice before UV exposure reduced UV-induced DNA damage, immunosuppression, inflammation, and sunburn cells [84], indicating that reduced PARP activity has an acute photoprotective effect. The current data support this interpretation from these studies.

The current study used a novel whole-cell ELISA-based assay to allow measurement of pADPr levels in primary human keratinocytes after UV exposure. Optimal PARP activity relies on a balance between pADPr accumulation and removal [69]. The data presented here support the proposal that 1,25(OH)_2_D_3_ increases energy availability and thus increased DNA repair in keratinocytes after UV, at least in part through a reduction in phosphorylation of ERK and thus a reduction in excessive accumulation of UV-induced pADPr.

## 4. Materials and Methods

### 4.1. Cell Culture

Samples of skin removed at elective surgery were harvested after obtaining written informed consent from subjects or their parents/guardians, with approval from the University of Sydney Human Ethics Committee (Reference number 2015/063) and according to Declaration of Helsinki Principles. Keratinocytes were cultured from these skin explants as previously described [43,48]. Keratinocytes (passages 2–5) from at least three independent donors were used in all experiments.

### 4.2. Treatments

1,25(OH)_2_D_3_ (Sapphire Bioscience Pty Ltd., Sydney, Australia) was solubilised in spectroscopic ethanol (Merck, Darmstadt, Germany), and the vehicle was 0.1% (*v*/*v*) ethanol. The concentration of this compound was determined by a Nanodrop ND-1000 Spectrophotometer (Thermo Fisher Scientific, Wilmington, NC, USA). The 1,25(OH)_2_D_3_ was added immediately after UV exposure, as is our standard protocol [45,51]. 3-Aminobenzamide (3AB) was from Sigma Aldrich (St. Louis, MO, USA) and was used at a concentration of 1 × 10^−3^ M [57,83]. ERK activation inhibitor, peptide II (IC_50_ = 0.21 µM), which selectively binds to ERK2, preventing its interaction with MEK [93], was purchased from Calbiochem-EMD4 Biosciences (Gibbstown, NJ, USA). Both inhibitors were present during and after UV exposure.

### 4.3. UV Irradiation

UV irradiation (ssUV) was provided by an Oriel Sol1A^TM^ 94042A 450W solar simulator (Newport Corporation, Irvine, CA, USA). The output of this lamp was around 10% UVB and 90% UVA as previously described [45]. Our standard protocol for irradiation used 400 mJ/cm^2^ UVB and 3600 mJ/cm^2^ UVA (4000 mJ/cm^2^ = Dose 1), measured by an IL1700 research radiometer (International Light Technologies, Peabody, MA, USA), equivalent to 4 min of noon-day sun in October in Sydney, Australia [43,51]. pADPr experiments were carried out at 3 times this level to test for significant increases in UV-induced pADPr levels.

### 4.4. PARP Measurements by Novel Whole-Cell ELISA

A UV-induced increase in PARP activity was used as positive control, as has been previously established to occur in skin cells [83]. 3-Aminobenzamide (3AB) was added to these cells to provide a negative control and was present during and after irradiation, as is standard protocol [57]. Cells were fixed with 10% (*v*/*v*) formaldehyde in PBS, pH 7.4, at 5 min and 15 min post-irradiation and treatment. Cell membranes were permeabilised with 0.1% (*v*/*v*) Triton X-100, and endogenous peroxidase activity was blocked with 1% (*v*/*v*) H_2_O_2_. Non-specific antibody binding was inhibited by 0.05% (*w*/*v*) heat-denatured casein (HDC) in PBS, pH 7.4, and pADPr was detected by incubation of mouse monoclonal IgG_3_ pADPr Antibody (10H): sc-56198 antibody (Santa Cruz Biotechnologies, Dallas, TX, USA) at 5 µg/mL in HDC overnight at 4 °C. Isotype mouse control antibody was used at a similar experimental concentration and under similar conditions. The following day, cells were incubated with anti-mouse IgG horse radish peroxidase (HRP)-linked antibody (Cell Signaling Technology, Danvers, MA, USA) at 0.4% (*v*/*v*) followed by addition of the HRP substrate, 3,3′,5,5′-tetramethylbenzidine (0.1 mg/mL in 0.05 M citrate 0.1 M phosphate buffer at pH 5.5) (Sigma-Aldrich, St. Louis, MO, USA) with 0.02% (*v*/*v*) H_2_O_2_. The colourimetric reaction was stopped by the addition of 50% (*v*/*v*) of 2 M H_2_SO_4_ (VWR international, Radnor, PA, USA). The absorbance was quantified at 450 nm. The absorbance generated by matched isotype wells was subtracted from test data to account for non-specific binding of the antibody.

### 4.5. CPD and 8-OHdG Immunohistochemistry

As an index of CPD, thymine dimers [94] and 8-OHdG were detected and measured as previously described [47,95]. In brief, primary human keratinocytes were cultured at 90% confluence on 5 mm poly-l-lysine-coated glass coverslips, exposed to UV and treatments as indicated and fixed at 1.5 or 3 h after UV (as stated). After rinses with phosphate buffered saline (PBS), cells were fixed with ice-cold 100% methanol (Sigma-Aldrich, St. Louis, MO, USA) at −20 °C for 10 min. Keratinocytes were then extensively washed with MilliQ water at pH 7.2 (Millipore SAS, Molsheim, France) and air dried at room temperature overnight. The next day immunohistochemistry was carried out as previously reported [43], with minor modifications. Treatment with peroxide (1% *v*/*v*—ThermoFisher Scientific, Waltham, MA, USA) in PBS for 5 min, followed by two MilliQ water washes at room temperature, was used to block endogenous peroxidase activity. Several steps were used for antigen retrieval. Sodium hydroxide (70 mM) (Sigma-Aldrich, St. Louis, MO, USA) in 70% ethanol for 2 min at room temperature was used to denature nuclear DNA. Protein digestion was carried out using proteinase K (Sigma-Aldrich, St. Louis, MO, USA) (1 μg/mL in 0.1 mM CaCl_2_) for 5 min for CPD detection and 10 min for 8-OHdG detection, followed by two MilliQ water washes. Treatment of cells with 50% (*v*/*v*) horse serum (Sigma-Aldrich, St. Louis, MO, USA) in PBS, pH 7.2 (blocker), for 1 hour at room temperature was used to inhibit non-specific antibody binding. Keratinocytes were incubated with primary antibody in blocker for 60 min at room temperature: mouse monoclonal anti-thymine dimer at 5 µg/mL (Sigma-Aldrich, St. Louis, MO, USA) was used for CPD or primary mouse monoclonal IgG2b anti-8-hydroxy-guanosine antibody (Santa Cruz Biotechnologies, Dallas, TX, USA) for 8-OHdG at 2.5 µg/mL or an equivalent concentration of isotype control, followed by three TBS-T washes at room temperature. Cells were incubated with biotinylated goat anti-mouse IgG secondary antibody (ThermoFisher Scientific, Waltham, MA, USA) diluted to 1:500 in Tris-buffered saline with 0.1% Tween 20 (TBS-T) for 15 min, followed by three TBS-T washes at room temperature. Streptavidin-conjugated horseradish peroxidase (HRP) (Invitrogen, Waltham, MA, USA) diluted 1:150 in TBS-T was added for 15 min, followed by three TBS-T washes to amplify the primary antibody signal. Insoluble HRP substrate, Diaminobenzidine (DAB) (Enhanced Liquid substrate System for Immunohistochemistry, Sigma-Aldrich, St. Louis, MO, USA), was then added for 5 min, followed by two MilliQ water washes following sufficient colour development. Isotype antibodies were used as controls for all experiments. Isotype controls resulted in minimal staining as previously reported [96]. Entellen rapid mounting medium (Merck, Darmstadt, Germany) was used to mount air-dried coverslips on glass slides for image analysis [43].

Bright-field images were acquired at 20× magnification on an Olympus stereo investigator scope (MBF Bioscience, Williston, VT, USA) or the Zeiss Axio Scan-Z1 slide scanner (Carl Zeiss Microscopy, Jena, Germany). Staining intensity was quantified using ImageJ software version 1.52u (released 17 March 2020) [47,95].

### 4.6. Statistical Analysis

All experiments were repeated a total of three times with triplicate measurement for each treatment group in each independent experiment. All experiments yielded similar results. Primary keratinocytes were sourced from different donors for replicate experiments. Generally, statistical analyses were carried out using GraphPad Prism version 8.3.1 released on 19 January 2020 (San Diego, CA, USA) by one-way ANOVA with Tukey’s post-test comparison unless otherwise stated.

## Data Availability

The data that support the findings of this study are available from the corresponding author upon reasonable request. Some data may not be made available because of privacy or ethical restrictions.
